# Comparative exploration of the carotid body in domestic animals: morphology, physiology, histology, and pathology

**DOI:** 10.3389/fvets.2024.1409701

**Published:** 2024-11-22

**Authors:** Semzenisi Ecaterina, Dragos Hodor, Ibrahima Mamadou Sall, Corina Toma, Alexandru-Flaviu Tăbăran

**Affiliations:** Department of Anatomic Pathology, Faculty of Veterinary Medicine, University of Agricultural Sciences and Veterinary Medicine, Cluj-Napoca, Romania

**Keywords:** embryology carotid body, morphology carotid body, function carotid body, innervation carotid body, blood supply carotid body, pathology carotid body, carotid body tumors, etiology paragangliomas

## Abstract

The aim of the study was to present a review of the literature and research on the carotid body (CB) over the past years and update the latest findings. The purpose of this article is to present a general overview and comparative analysis of CB between species, from the microanatomy to the pathology of CB. This study gives information about the embryological development and physiological aspects of anatomical findings and their differences. The second part of the article gives a comparative analysis of the pathology of CB. Neoplasia of the CB in humans, namely, paraganglioma, in most cases, is part of a genetic MEN syndrome (multiple endocrine neoplasia). In dogs, paraganglioma is also involved with multiple neoplasia formations throughout the body, including endocrine and neuroendocrine glands. From this perspective, dogs are the most suitable specimens for studying carotid body tumors and their involvement in a MEN-like syndrome.

## 1 Introduction

For many years, the carotid body has been a significant and intriguing subject of research, drawing on insights from a range of disciplines including anatomy, physiology, and pathology. This interdisciplinary approach, which underscores the complexity and depth of the subject, has led to a deeper understanding of its morphology, physiology, histology, and pathology. The carotid body, also known as the “glomus caroticum,” “inter-carotid,” “glandula carotica,” and “carotid ganglion,” is a complex and multifaceted area of study that spans multiple fields of research.

The presence and localization of this structure have long been known in the scientific community. However, the first person who was able to describe the structure elegantly was De Castro in his publication (1). This study provided the first comprehensive description of the carotid body’s structure and innervation, highlighting the organ’s rich vascular supply and the complex network of sympathetic axons and glandular cells. Building on this foundation, in 1928, De Castro published “Nouvelles contributions à la connaissance du ganglion intercarotidien” in the same journal (2). This study offered a more detailed examination of the innervation of the carotid body and carotid sinus, confirming the presence of distinct sensory receptors, including chemoreceptors and baroreceptors. His findings helped resolve ongoing scientific debates regarding the sensory nature and physiological role of the carotid body. This was the theme of De Castro’s life, to which he devoted more than half of his scientific career. At the same time, in the scientific field, Hering has been studying the inter-carotid branch and discovered the Hering’s nerve (a branch of the glossopharyngeal nerve) that innervates the carotid sinus (3, 4). His experiments showed that the carotid sinus has pressure-sensitive receptors (baroreceptors) crucial in regulating arterial blood pressure. Hering was initially involved in the debate over whether the carotid body had a sensory function. While his primary focus was on the baroreceptors of the carotid sinus, he also recognized the importance of the nearby carotid body in chemoreception, which was later thoroughly explored by De Castro and Heymans (163, 164). Parallelly, Heyman carried out his research on the phenomenon of vasoconstriction of the cardio-aortic region. Heymans demonstrated in his work in 1927, “Sur les modifications directes et sur la régulation réflexe de l’activité du centre respiratoire de la tête isolée du chien” (5), that the carotid body plays a crucial role in detecting changes in blood chemistry, such as oxygen and carbon dioxide levels, and in regulating respiratory responses. Heyman had interacted with De Castro’s anatomical studies before the Civil War. Due to the Spanish Civil War and broader European conflicts in 1936, De Castro was isolated from scientific activity, which subsequently distanced him from in-depth scientific research. The collaboration between Heymans and De Castro helped establish the carotid body as a key organ in the reflex control of respiration, a discovery that eventually contributed to Heymans being awarded the Nobel Prize in Physiology or Medicine in 1938 (6, 7, 165).

CB is a crucial part of the chemoreceptor organs (alongside the aortic bodies) (8). It is situated at the bifurcation of the common carotid artery embedded in the adventitia. It is composed of type I cells and type II cells, which become excited by sympathetic and parasympathetic nerve fibers and release norepinephrine and acetylcholine. These characteristics give the carotid body the role of a polymodal sensor with complex functions for measuring pressure and monitoring variations of pH, pCO_2_, and pO_2_ in the blood. Additionally, it plays a crucial and intriguing part in some renal and hormonal processes in the body, sparking curiosity about its role. In the last decade, important discoveries have been made, such as the involvement of CB in neurogenesis, which may open prospects for the fight against Parkinson’s disease and the treatment of neurogenic hypertension, congestive heart failure, inflammatory responses, and obstructive sleep apnea. Some animal species, such as dogs, are ideal examples for studying the formation of paragangliomas, which have again aroused the interest of the scientific community. This microscopic “orchestra” plays a crucial role in the whole biochemical machinery, a fact that continues to intrigue researchers and veterinarians in the field (4).

## 2 Methods

### 2.1 Literature search

The literature search followed the Preferred Reporting Items for Systematic Reviews and Meta-Analyses (PRISMA) guidelines (166). The data were systematically searched in the following databases: Web of Science (“All databases” selected), PubMed (“All databases” selected), and SpringerLink (last accessed on 26 October 2023), using the keywords “embryology carotid body,” “morphology carotid body,” “structure carotid body,” “function carotid body,” “innervation carotid body,” “blood supply carotid body,” “pathology carotid body,” “carotid body tumors domestic animal,” “etiology paragangliomas.” Eligibility criteria: research articles and book chapters. Information sources: databases and manuals (book chapters). The study selection process was performed by screening (e.g., the abstract). The data were searched separately by two of the researchers (Semzenisi Ecaterina and Ibrahima Mamadou Sall). A total of 2,657 duplicates were excluded. Another 794 results were excluded because of other reasons: “embryology carotid body” not found (*n* = 7), “morphology carotid body” not found (*n* = 172), “function carotid body” not found (*n* = 197), “innervation carotid body” not found (*n* = 68), “blood supply carotid body” not found (*n* = 98), “carotid body tumor domestic animal” not found (*n* = 16), “pathology carotid body” not found (*n* = 108), “etiology paragangliomas” not found (*n* = 71). Furthermore, reviewed articles were excluded (*n* = 1,321). The remaining articles (*n* = 425) were included in the current study, and the data were further extracted and analyzed.

## 3 Anatomical and physiological aspects of CB

### 3.1 Embryogenesis

According to Hempleman and Warburton (5), the cephalic reorganization was the first step in the evolution of fish, amphibians, birds, and mammals. The development of articular jaws for capturing prey was a crucial step, as well as the evolution of circulatory and respiratory systems, providing an active way of life. These changes required the development of all paired organs, including CB. During embryogenesis, CBs are derived from the neural crest, namely from neural stem cells (pluripotent cells) and neural progenitor cells (sympathetic neuroblasts). Consequently, these cells migrate to various parts of the embryo and give rise to several cell types: sympathetic neurons, Schwann cells, adrenal chromaffin cells, and some paired cranial sense organs, including those forming the CBs. Gans and Northcutt, in their work “Neural Crest and the Origin of Vertebrates 1983,” suggest in their theory that neural crest cells and epidermal placodes played key roles in this transformation. Namely, epidermal placodes are controlled by the development of the neck and head. They create key sensory organs such as the eyes, ears, and nose, as well as the sensory ganglia associated with many cranial nerves. Consequently, the inferior ganglion of the vagus (X) nerve and petrosal ganglia of the glossopharyngeal nerves (IX) have double origin from epidermal placodes and neural crest cells. They provide innervation in avian and mammalian CB embryogenesis. These arise from the third pharyngeal arch (branchial arches in aquatic species) (5–8, 10).

In embryological development in mammals, CB arises from the superior cervical ganglion and takes root at the wall of the carotid artery. One of the latest studies regarding the intrauterine evolution and function of CB suggests that during the intrauterine period, CB has an endocrine function. However, in the postnatal period, this organ acquires the chemoreceptor function (5, 6, 11, 12).

In humans, glomus cells can be detected starting from 13 weeks of pregnancy (total period 38–42 weeks), and at 14–16 weeks, they are remarkably mature compared to adult cells. They present many anastomoses and nerve packages, and they are constantly separated from the blood vessel walls (6, 7, 10).

In rabbits, the first detection of glomus cells in the fetus begins at E13 days of gestation (normal gestation 21–24 days). At 13.5–14 days, the glomus cells are quite mature, and they can be differentiated into two types of cells. Type I cells are more developed, with a pronounced nucleus and Golgi apparatus. Type II cells are less developed and located at the periphery. After 3 days, a rich network of unmyelinated nerve fibers can be observed (13). In rats, the development begins with the transformation of neural stem and progenitor cells. These cells are involved in central nervous system development which are transformed to sympathetic neuroblasts around E12 (embryonic day), followed by cellular differentiation around 13.5 × E14 and vascularization around E16. By late gestation day 20, the carotid body achieves its mature structure, and its functional capabilities continue to develop postnatally (11, 14).

In mice, the carotid body begins with the migration of neural progenitor cells around day E9.5, so it generates distinct neuronal and glial subtypes over time, followed by cellular differentiation around embryonic day 11.5 and vascularization around embryonic day 13.5. By late gestation day 17.5, the carotid body achieves its mature structure. However, transgenic mice lacking Mash-1 (also known as Ascl1) do not develop sympathetic ganglia or glomus cells (Type I), but sustentacular (Type II) cells are present (discussed in morphology and structure) (15–17).

In the birds, CB is located in the cervicothoracic border, at the beginning of the subclavian and common carotid arteries. CB in embryos can be first observed at 6–8 days of development (total fetal period: 21 days). Unlike mammals, the genomic cells migrate from the distal vagal ganglion during the development of the fetus. This migration phase is caused by cervical elongation (13, 18). Then, in adult birds, we can observe a linear arrangement of the carotid body, thyroid gland, parathyroid glands, and ultimobranchial gland, arranged in a continuous line along the common carotid artery. The left side of this arrangement includes the carotid body, two parathyroid glands, and the ultimobranchial gland, while on the right side, the complex is adjacent to the lower part of the thyroid gland (10, 13, 18).

CB in reptiles begins with neogenesis, the precursor of gliogenesis, similar to other vertebrates. These cells migrate and form the carotid body primordium in the wall of the third pharyngeal arch artery. Various studies have provided both direct and indirect evidence for the presence of cardiorespiratory chemoreceptors at key vascular sites in reptiles, including lizards. The chemoreceptive cells identified in tegu lizards are homologous to the glomus cells found in mammals, turtles, and snakes, suggesting a conserved evolutionary mechanism for detecting changes in blood chemistry and regulating vascular tone (19, 20).

Amphibians possess a structure called the carotid labyrinth at the origin of the internal carotid artery, which serves a similar function but has a distinct morphological arrangement compared to the typical carotid body in other vertebrates. The carotid labyrinth in amphibians shares functional similarities with the carotid body in other vertebrates, particularly in its role in monitoring blood flow dynamics. In fish, oxygen-sensitive chemoreceptors are distributed along various respiratory structures. These include *Orobranchial Cavity*: it found within the walls of the orobranchial cavity and innervated by cranial nerves V (trigeminal) and VII (facial). *Spiracle and Pseudobranch*: in species possessing these structures, chemoreceptors are located here and are innervated by cranial nerves VII (facial) and IX (glossopharyngeal) (13, 20, 167) (Table 1).

**Table 1:** Development and aging of the carotid body (CB) across different species.

Species	Gestational age/period	First appearance of CB as an organ	Differentiation of type 1 and type 2 cells	Developmental highlights	Aging highlights of CB in adult species
Humans	38–42 weeks	7 weeks	13 weeks	At 14–16 weeks, glomus (Type I) cells and sustentacular (Type II) cells are present and mature. At 7 weeks, CB is in close contact with the internal carotid artery and sympathetic trunk anlage. At 11–16 weeks, CB is a separate organ from the carotid artery and sympathetic trunk. At 13 weeks, sustentacular and glomus cells can be distinguished.	Reduction in the number of oxygen-sensing cells, decreased mitochondrial content, increased extracellular matrix, reduced synaptic contacts, and lower expression of key hypoxia-responsive proteins. These alterations collectively result in a diminished hypoxic ventilatory response and an overall decline in the chemosensory function of the carotid body.
Rat	21–23 days	13.5 days	14 days	At 13.5 days, thickening of the arterial wall with undifferentiated cells occurs. At 14 days, presumptive glomus (Type I) cells and other cell types are seen. At 17 days, CB is separated from the artery wall and surrounded by a capsule. Undifferentiated cells (possible sustentacular (Type II) cell progenitors) are present.	Similar to humans.
Rabbit	30–33 days	13.5 days	14 days	Developmental events are similar to rats but with a slower time course.	–
Mouse	19–21 days	11.5 days	11.5–12.5 days	CB primordia form when cells from the superior cervical ganglion enter the wall of the third aortic arch. Transgenic mice lacking Mash-1 do not develop sympathetic ganglia or glomus cells (Type I). However, sustentacular (Type II) cells are present.	–
Bird (chicken)	21 days (hatching at 21 days)	6–8 days	12 days	At 6–8 days, CB anlage is discernible in the lateral portion of the third branchial arch arteries. - At 12 days, differentiation of glomus (Type I) and sustentacular (Type II) cells occurs. - Nerve fibers derived from vagal ganglia penetrate CB primordium by 12 days and increase to adult density by 18 days.	–

Adapted from (174).

### 3.2 Normal morphology and structure

**Figure 1:**
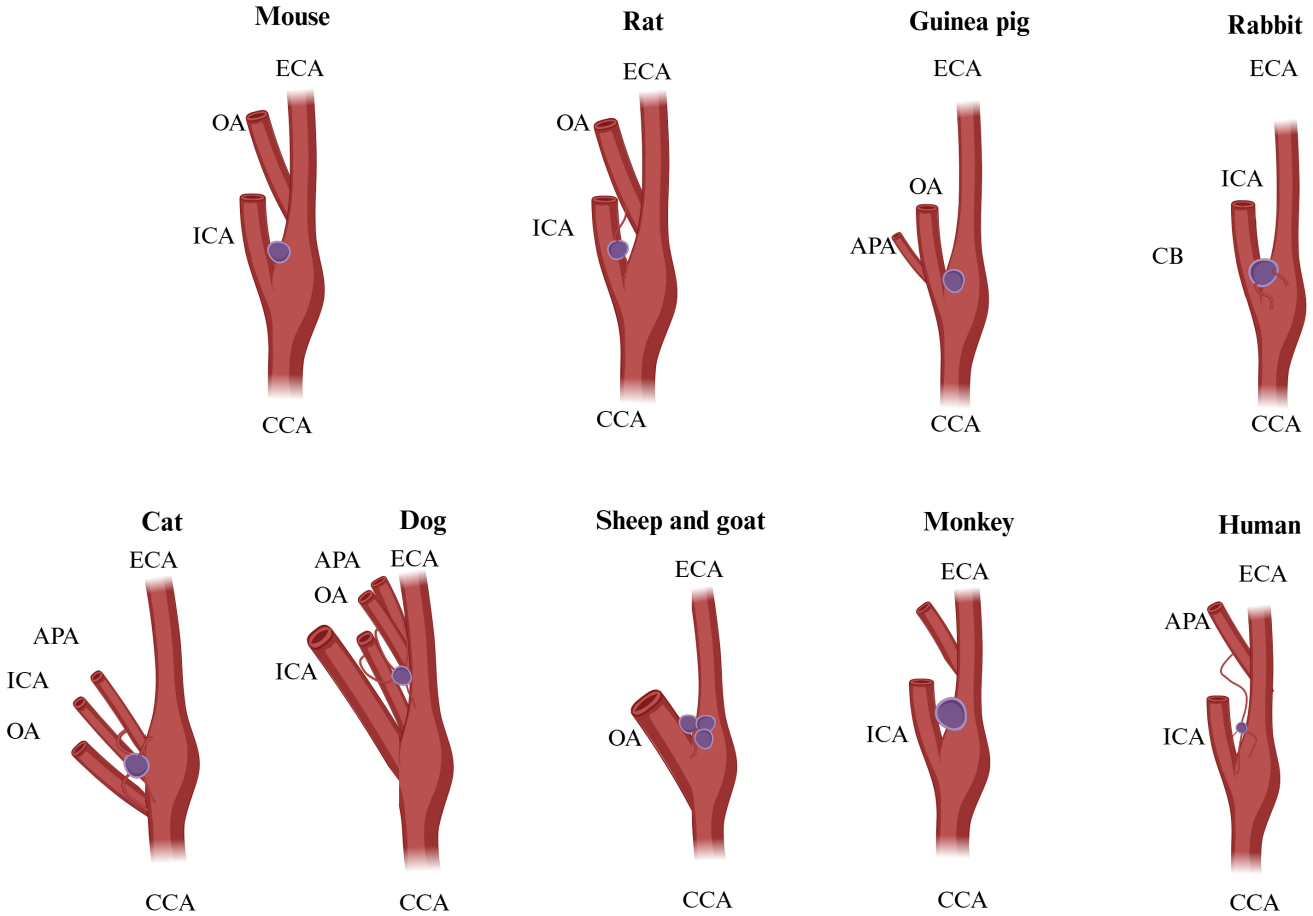
Possible origins of CB blood supply in different species. Mouse: origin of vascularization not known. Rat: ECA, external carotid artery; OA, occipital artery. Guinea pig: not known. Rabbit: vagus X bifurcation. Cat: OA, occipital artery; APA, ascending pharyngeal artery; ECA, external carotid artery. Dog: OA, occipital artery; APA, ascending pharyngeal artery; ECA, external carotid artery. Sheep and goat: OA, occipital artery. Monkey: not known. Humans: ECA, external carotid artery; ICA, internal carotid artery; APA, ascending pharyngeal artery. Adapted from Brognara et al. (130).

Although CB is a paired organ, slight asymmetry can be observed between the two sides. The position of the CB may show variations depending on the species, size, breed, and specific anatomical features (Figure 1) (10, 21). Structurally, CB is composed of multiple lobules and well-organized clusters (also named glomeruli). CB lobules are separated by fine bundles of well-vascularized fibrous connective tissue, named septal walls, which derive from the capsule and constitute the stroma of the organ. The amount of connective tissue increases with the age of the animal but generally accounts for approximately 50–60% of the total volume of the organ (Table 1). Accordingly (21, 22), aging affects the carotid body in both humans and rats by decreasing its sensitivity and functional capacity, largely due to structural and molecular alterations. Aging and chronic hypoxia of CBs showed increased cytoplasm/mitochondria volume ratios and reduced numbers of glomus cells, secretory vesicles, and mitochondria. There was also increased extracellular matrix and fibronectin, indicating fibrotic changes. Resulting in a gradual loss of the homeostatic function. Current literature lacks data demonstrating the effects of aging on the carotid body in birds (22–24).

**Figure 2:**
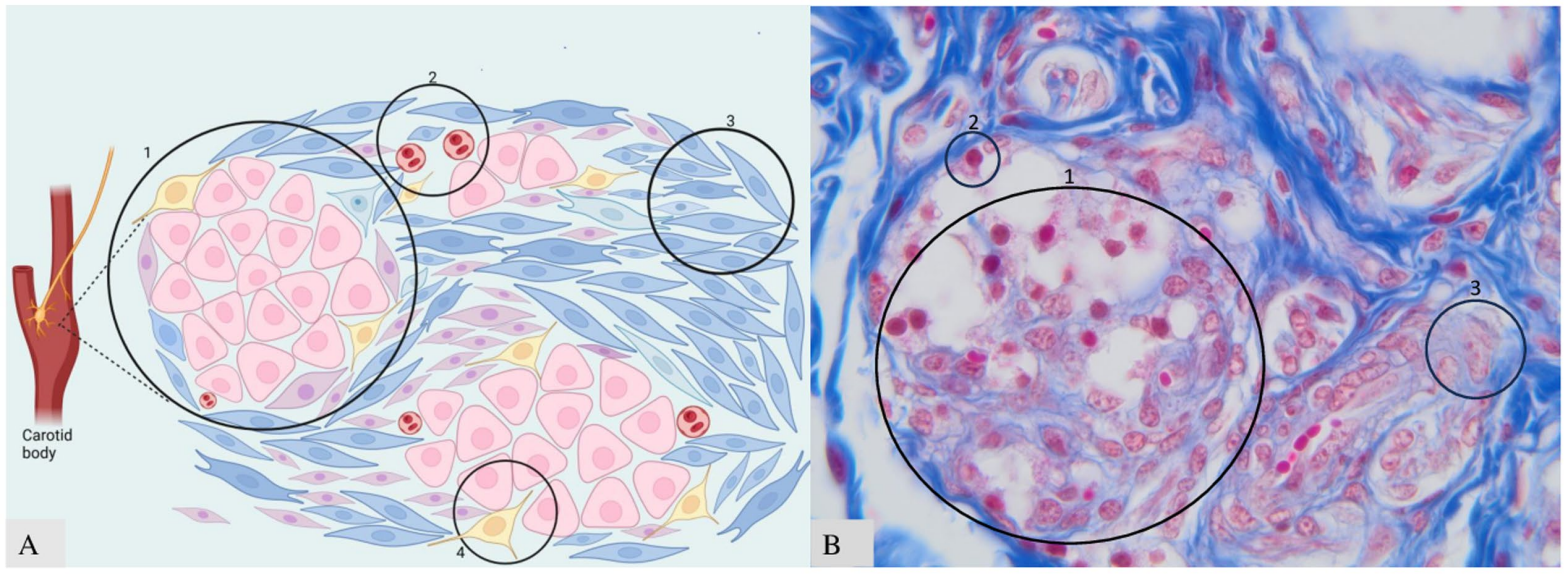
Clusters of neuroendocrine cells in CB. (A) Schematic representation: A—type I in the center, type II at the periphery; B—blood vessels; C—fibrous connective tissue; D—nerve fibers. (B) Histology of CB with three components visible. B—microscopic appearance of clusters and glomus cells 40× scale bar = 200 μm. Adapted from Clarke et al. (25) and Atanasova et al. (12).

The essential structure of the CB includes four basic components (Figure 2): (1) clusters of neuroendocrine cells (organized as glomoids or glomeruli, considered the functional part of the carotid body), separated by (2) blood capillaries and supported by (3) fibrous connective tissue enriched by (4) sympathetic and parasympathetic nerves. Within each lobule, the clusters are composed of variable numbers of neuroendocrine cells (i.e., 2–12 cells in rats), which are stimulated by adrenaline (epinephrine), noradrenaline (norepinephrine) T4, and cortisol. All these hormones regulate stress activity, metabolism, energy balance, and cardiorespiratory rates (25).

The morphological unit of CB includes two types of cells named: “Cells type I” and “Cells type II” (Figure 2). These cells have neuroendocrine functions. Neuroendocrine cells have many features that are similar to those of neurons and endocrine cells. The main common appearance is represented by the presence of vesicles within the cytoplasm containing the “synaptophysin” protein and secretory granules containing the protein “chromogranin,” which can be found in paraganglia derived from the neural crest. It was demonstrated that a small number of cells were found around the common carotid artery—named “Miniglomera”—like glomus cells (26, 27).

Glomus cells (type I cells) are the primary chemoreceptive elements in the CB, responsible for detecting changes in blood oxygen (pO_2_), carbon dioxide (pCO_2_), and pH levels. Upon sensing hypoxia, hypercapnia, or acidosis, these cells depolarize due to the inhibition of potassium channels. This depolarization leads to the opening of voltage-gated calcium channels, resulting in an influx of calcium ions (Ca^2+^). The increase in intracellular calcium triggers the release of neurotransmitters such as acetylcholine (ACh), adenosine triphosphate (ATP), and dopamine from dense-cored vesicles. Such a recent finding was to establish the distribution of dopamine D2 receptors in the CB of rats. The findings reveal that these receptors are present in the glomus cells, suggesting a role in chemoreception and potential regulation of respiratory functions (14, 28, 29).

In cases of hypoxia, this activates a variety of genes primarily through the stabilization and activity of hypoxia-inducible factors (HIFs). VEGF (vascular endothelial growth factor); promotes angiogenesis, EPO (erythropoietin; stimulates red blood cell production, GLUT1 (glucose transporter 1; increases glucose uptake), LDHA (lactate dehydrogenase A; supports anaerobic metabolism), BNIP3 (BCL2-interacting protein 3; involved in apoptosis). These genes help cells to adapt to low oxygen conditions by improving oxygen delivery and optimizing metabolic pathways (30, 31).

Type I cells (initially called periadventitial type I cells) are located in the center of CB lobules, but rare, irregular, clusters of type I cells can be found adjacent to internal and external carotid arteries, embedded in the connective tissue (21, 25, 32). The cells type I have a round\ovoid shape, and they are situated in the inner part of CB, interconnected with a rich capillary network. They are also called “chief cells” and contain dense-core synaptic vesicles that are positive for tyrosine hydroxylase (TH). TH is an enzyme crucial for metabolizing catecholamines, including dopamine, norepinephrine, and epinephrine. It catalyzes the conversion of tyrosine to L-DOPA, which is the rate-limiting step in producing these neurotransmitters. A recent study demonstrated that short-term hypoxia increases TH immunoreactivity in rat CB and plays a role in the chemosensory function (31).

There are two vesicles: celear core vesicles and dense core vesicles. Clear core vesicles, also known as synaptic vesicles, are presumed to store non-catecholamine neurotransmitters like acetylcholine. They are generally about 40 nm in diameter and dispersed throughout the cytoplasm. Clear core vesicles, also known as synaptic vesicles, are typically found in neurons and are involved in the storage and release of neurotransmitters at synaptic junctions. In the context of the CB, these vesicles play a crucial role in the neurotransmission process between glomus cells and afferent nerve fibers (14, 29, 31).

However, dense core vesicles are larger, ranging from 50 to 150 nm in diameter, and contain catecholamines (primarily dopamine), chromogranin neuropeptides, adenine nucleotides, and Ca^2+^. These vesicles are crucial for the chemosensory function of type I cells. Neuroendocrine characteristics: Type I cells share similarities with adrenal medulla chromaffin cells, but they do not depend on nerve growth factors or corticosteroids for survival in culture. Instead, they rely on basic fibroblast growth factor 2 (FGF2) for mitogenesis and survival (14, 31). Another very valuable research regarding tyrosine hydroxylase (TH) was carried out in the rat CB. The study concludes that the CB, like the vagal paraganglia, plays a significant role in the immune-to-brain communication pathway. The upregulation of interleukin-1 receptor in type I cells (IL-1RI) and TH in the CB following IL-1 stimulation suggests that these glomus cells are responsive to pro-inflammatory cytokines. This responsiveness provides morphological evidence that contributes to the hypothesis that the CB can even detect and transmit peripheral immune signals to the central nervous system, contributing to the overall understanding of the interaction between the immune system and the brain during systemic inflammation (32).

In 2023, Dr. Villadiego and his team presented significant findings in their publications and research, advancing our understanding of neurotrophic factors and their applications in Parkinson’s disease therapy. The protein anosmin-1 (A1) regulates the development and survival of dopaminergic neurons in different nuclei of the mammalian nervous system. Transgenic mice present smaller CB with a reduced number of dopaminergic chemo-sensitive glomus cells. Moreover, A1 significantly increases the number of tyrosine hydroxylase-positive TH^+^ dopaminergic neurons in the CNS, particularly in the substantia nigra pars compacta (SNpc), but does not alter susceptibility to MPTP-induced parkinsonism. 1-methyl-4-phenyl-1,2,3,6-tetrahydropyridine is a neurotoxin that is used in scientific research to create animal models of Parkinson’s disease. Stem cell-derived CB glomus cells provide neuroprotective and neurorestorative effects similar to native CB tissue in a chronic (MPTP) model of Parkinson’s disease. These cells represent a promising therapeutic option for cell-based treatments in PD, capable of protecting nigral neurons and promoting the regeneration of dopaminergic pathways through the sustained release of GDNF (glial cell line-derived neurotrophic factor). Following this, CB cell therapy has been proven effective in rodent and non-human primate preclinical Parkinson’s disease (PD) models. The beneficial effects are primarily due to GDNF released by grafted CB glomus cells. This creates encouraging horizons for future research and offers significant hope for the fight against Parkinson’s disease (16, 17).

Regarding type II, cells play a metabolic support role for the extracellular environment, influencing the activity and sensitivity of type I cells by maintaining ionic balance and removing metabolic waste products (14, 31, 32). They are spindle-shaped and are localized at the periphery of type I cells, providing sustentacular functions. The nuclei of type II cells are more chromatin-dense and flattened compared to the large, euchromatic nuclei of type I cells. These cells lack the dense-cored vesicles found in type I cells but possess typical organelles such as mitochondria and rough endoplasmic reticulum, indicating their supportive and metabolic roles. Type II cells exhibit characteristics like glial cells and glial-like functions. Correspondingly, the data indicate that they express proteins such as S-100, GFAP (glial fibrillary acidic protein), and DBH (dopamine β-hydroxylase). All these are crucial enzymes in the biosynthesis of catecholamines, making them important markers in diagnosing and understanding neuroendocrine tumors (23, 30, 33).

Type II cells are glial cells in the nervous system, providing structural and metabolic support. They represent approximately 30–40% of the total cell population of CB. These cells are non-excitable as they lack voltage-sensitive channels of Na^+^ and Ca^+^ but possess chemo-transduction and plastic functions. However, the functionality of these cells was underestimated. Recently, it was demonstrated that they have a paracrine role. Therefore, it was confirmed that their chemo-transduction and plastic functions (34, 35).

This process was so-called crosstalk between neighboring cells, underlying the biochemical processes triggered by several neurochemical elements: adenosine triphosphate (ATP), angiotensin II (ANGH II), endothelin-1, dopamine, histamine, and 5-hydroxytryptamine (5-HT), which is known to be expressed in chemoreceptor type I cells—they increase intracellular Ca^+^ in type II cells. The catabolization of ATP by ectonucleotidase creates another active ligand that interacts with pre- and postsynaptic receptors in the sensory synapse (168). This process is described as a chain reaction produced by type I cells, which activates the excitatory receptors to be placed on the surface of type II cells, which express ligand-gated purinergic signaling receptors; in other words, type II cells have the role of the vehicle for ATP induce and ATP release, following the activation of autocrine/paracrine signaling pathways during the chemo-transduction. This interaction ensures a coordinated cellular response to hypoxia, involving multiple genes and proteins that help the cells adapt to low oxygen conditions by promoting processes such as angiogenesis, metabolism adjustment, and survival mechanisms. However, the paracrine role of cells type II is not fully studied during different conditions and deviations of neurochemicals. Following this, it was determined that Cells type II plays a crucial role in creating a pathway through the petrosal ganglion to the nucleus tractus solitary (NTS). There are some hypothetical models for a potential role of type II, which might act as an enhancer in the release of a crucial excitatory neurotransmitter, ATP, through the involvement of purinoceptors P2Y and channel pannexin-1 (29, 36, 37, 169).

Recent research suggests that type II cells may act as progenitor cells, with the potential to differentiate into type I cells under certain conditions, such as chronic hypoxia. This plasticity is crucial for the carotid body’s adaptive response to prolonged low oxygen levels, allowing for an increase in the number of chemoreceptive type I cells when needed. All these facts highlight their potential importance beyond initial expectations (29, 38).

### 3.3 Functions

**Figure 3:**
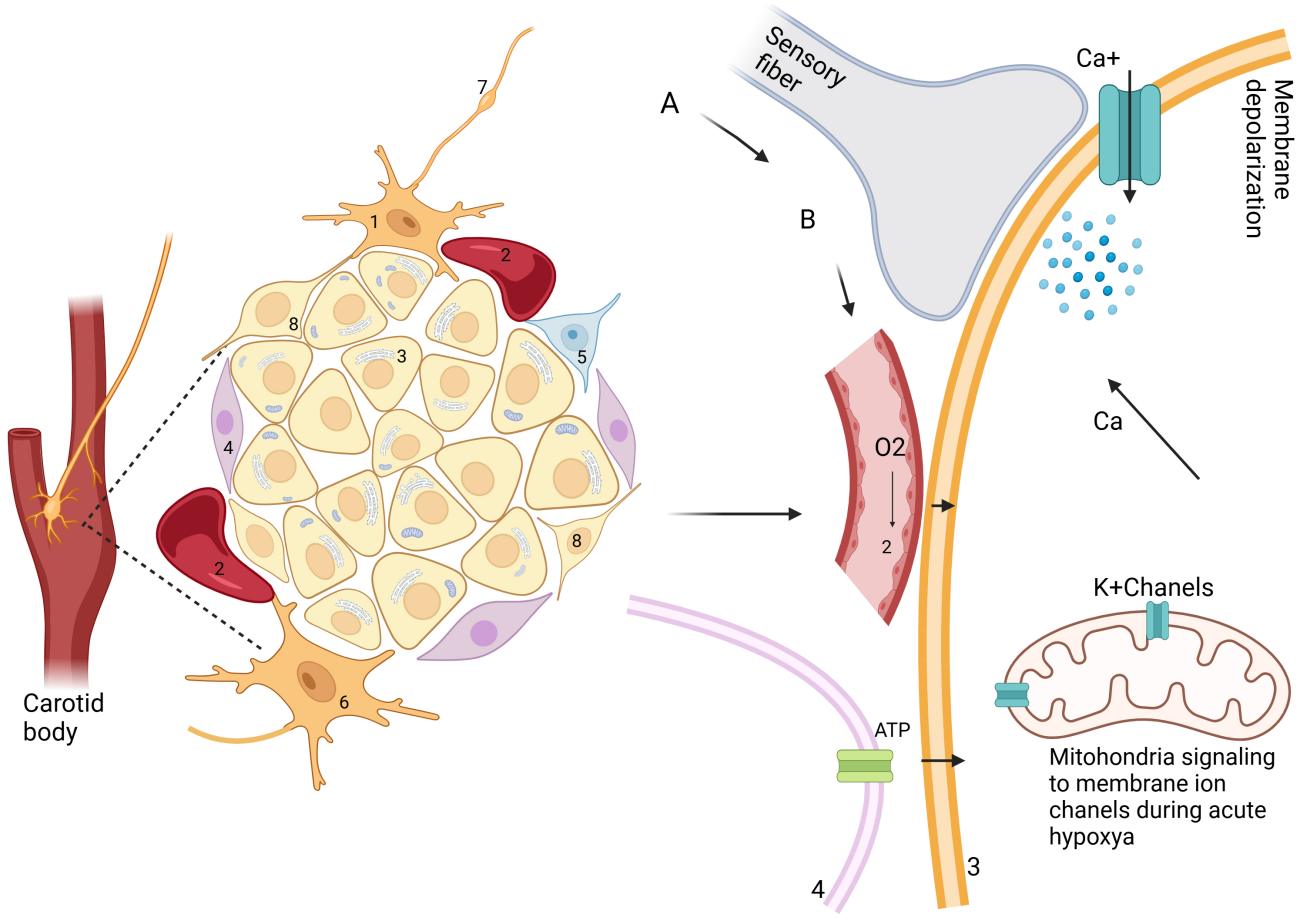
A schematic representation of the interaction between cell types I and II in CB. 1—afferent neuron; 2—blood vessels; 3—glomus cell (type I); 4—cell type II; 5—neuroblast cell; 6—efferent neuron; 7—glossopharyngeal nerve and petrosal ganglion; 8—progenitor cell. (A) Other stimuli: leptin, insulin, temperature, osmolarity, reduced blood flow. (B) hypercapnia, acidemia, hypoxia, hypoglycemia.

**Figure 4:**
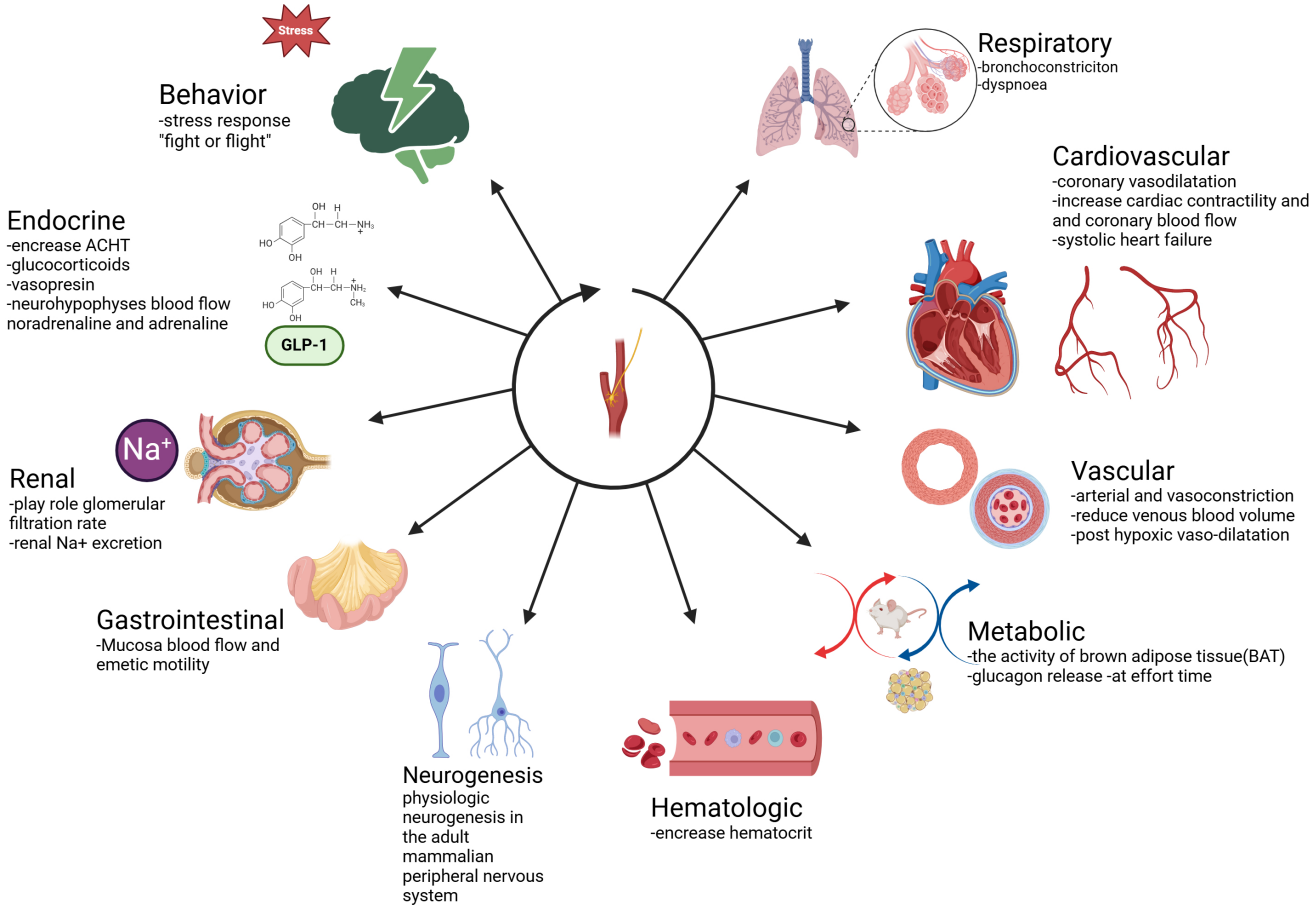
Functions of CB. Respiratory: bronchoconstriction, dyspnea. Cardiovascular: coronary vasodilatation—increase cardiac contractility and coronary blood flow—systolic heart failure. Vascular: arterial and vasoconstriction-reduce venous blood volume-post hypoxic vasodilatation. Metabolic: the activity of brown adipose tissue (BAT), glucagon release at effort time. Hematologic: increase hematocrit. Neurogenesis. Physiologic neurogenesis in the adult mammalian peripheral nervous system. Gastrointestinal: Mucosa blood flow and emetic motility Renal: plays role in glomerular filtration rate-renal Na^+^ excretion Endocrine: increase ACHT-glucocorticoid. Adapted from Zera et al. (129).

The main function of CB is to maintain homeostasis, influencing the level of blood pressure in arterial and venous flow. In addition, CB plays a crucial role in the respiratory, cardiovascular, gastrointestinal, renal, metabolic, hematologic, and endocrine activities (Table 2; Figures 3, 4). In the last decade, CB has gained huge scientific interest due to its polymodal functions, which contribute to systolic heart failure, obstructive sleep apnea, hypertension, and cardiometabolic diseases (14).

**Table 2:** Functions of CB.

Functions	Process
Respiratory	Bronchoconstriction (14, 131, 132). Dyspnea (133, 134).
Cardiovascular	Coronary vasodilatation (135). Increase coronary blood flow and cardiac (136). Contractility (134, 135, 137). Systolic heart failure (42).
Vascular	Arterial and vasoconstriction (138) Reduce venous blood volume (139) Splenic capsule contraction (140) Post hypoxic vaso-dilatation (140).
Renal	Play role glomerular filtration rate (141) Renal Na^+^ excretion (142) Obstructive sleep apnea (OSA) (141)
Metabolic	The activity of brown adipose tissue (BAT) (131, 132, 135) Glucagon release—at effort time (27)
Hematologic	Increase hematocrit (143)
Endocrine	Play role in increasing ACHT 52 Glucocorticoids (35) Vasopressin (35) Neurohypophyses in blood flow noradrenaline and adrenaline (144)
Gastrointestinal	Mucosa blood flow and emetic motility (145, 146)
Behavior	Fight or flight (129)
Neurogenesis	The carotid body contains stem cells that sustain physiologic neurogenesis in the adult mammalian peripheral nervous system (PNS) (17).

Adapted from Atanasova et al. (26), Gueguen et al. (28), and Kumar et al. (29).

### 3.4 Innervation

**Figure 5:**
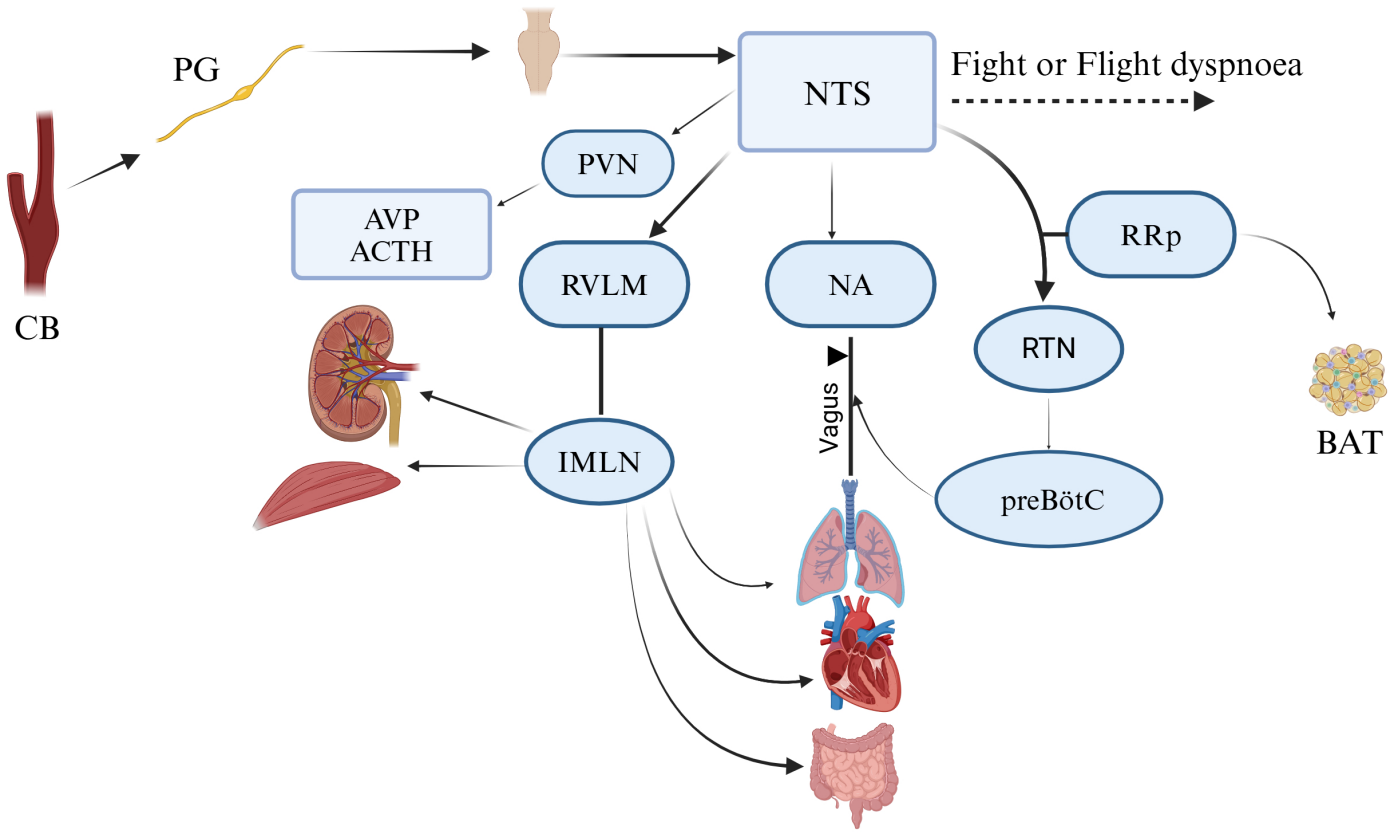
Neural communication of CB in mamalians. It creates a connection analogic to ribbon cable that arises from glomus cell and makes connecting with NTS (nucleus tractus solitarii); RVLM (rostral ventrolateral medulla); NA (nucleus ambiguous); PVN (paraventricular nucleus of the hypothalamus); RRp (nucleus rostral raphe pallidus); IMLN (intermediolateral nucleus); RTN (retro-trapezoid nucleus); preBötC (ventral respiratory group); AVP (arginine vasopressin); ACTH (adrenocorticotropic hormone); BAT (the activity of brown adipose tissue). Adapted and modified from Zera et al. (129).

The first important neural unit is “plexus intercaroticus,” which is formed by many ramifications which arise from the main cervical ganglion. Arising from the vagal trunk and glossopharyngeal nerve, they play a crucial role in parasympathetic innervation of the head and neck, including the lacrimal gland, nasal glands, and palatine glands, as well as the taste and vasomotor functions of the nasal region and CB (39, 40). Although the innervation of the CB is carried out by various centrifugal fibers: postganglionic vasomotor, related to the sympathetic nervous system from the superior cervical ganglion via ganglion-glomerular nerve (plays a role in the regulation of blood pressure and kidney function through to the secretion of renin and regulation of sodium reabsorption). These postganglionic neurons have the function of innervating just blood vessels; however, we can find these cells outside, some of them inside, and alongside the CB (41). From this plexus, fibers come to the organ and create numerous connections inside, forming a network of fibrils and bringing information to the brainstem (Figure 5) (4, 39). All the information collected by sympathetic and parasympathetic systems is delivered to the Nucleus Tractus Solitaire (NTS). It is the main nucleus, which divides the impulses received from the sensory baroreceptors to other brain centers. If we take a look at the structure and position of sympathetic and parasympathetic neurons, they are located circumferentially within the CB. Autonomic innervation of microcirculation is not well described. McDonald suggests that the glomic capillaries can be involved in vasomotor changes by the straight effect of extensive multifunctional mural cells, known also as pericytes (29, 42). After 10 years, it was confirmed that glomic capillaries are surrounded by an abundant number of pericytes and sustentacular cells, arranged in a concentric or spiral position, interconnected with the sustentacular glomic cells, having direct hyperplastic responses to systemic hypertension and chronic hypoxemia (Figure 5) (41). In birds, the carotid bodies receive innervation from fine nerve branches of the adjacent vagus nerve (cranial nerve X), vagal nodose ganglia, vagal recurrent nerves, and from the sympathetic trunk (specifically at the level of the 14th cervical sympathetic ganglion in chickens) (11, 18).

### 3.5 Blood supply

**Figure 6:**
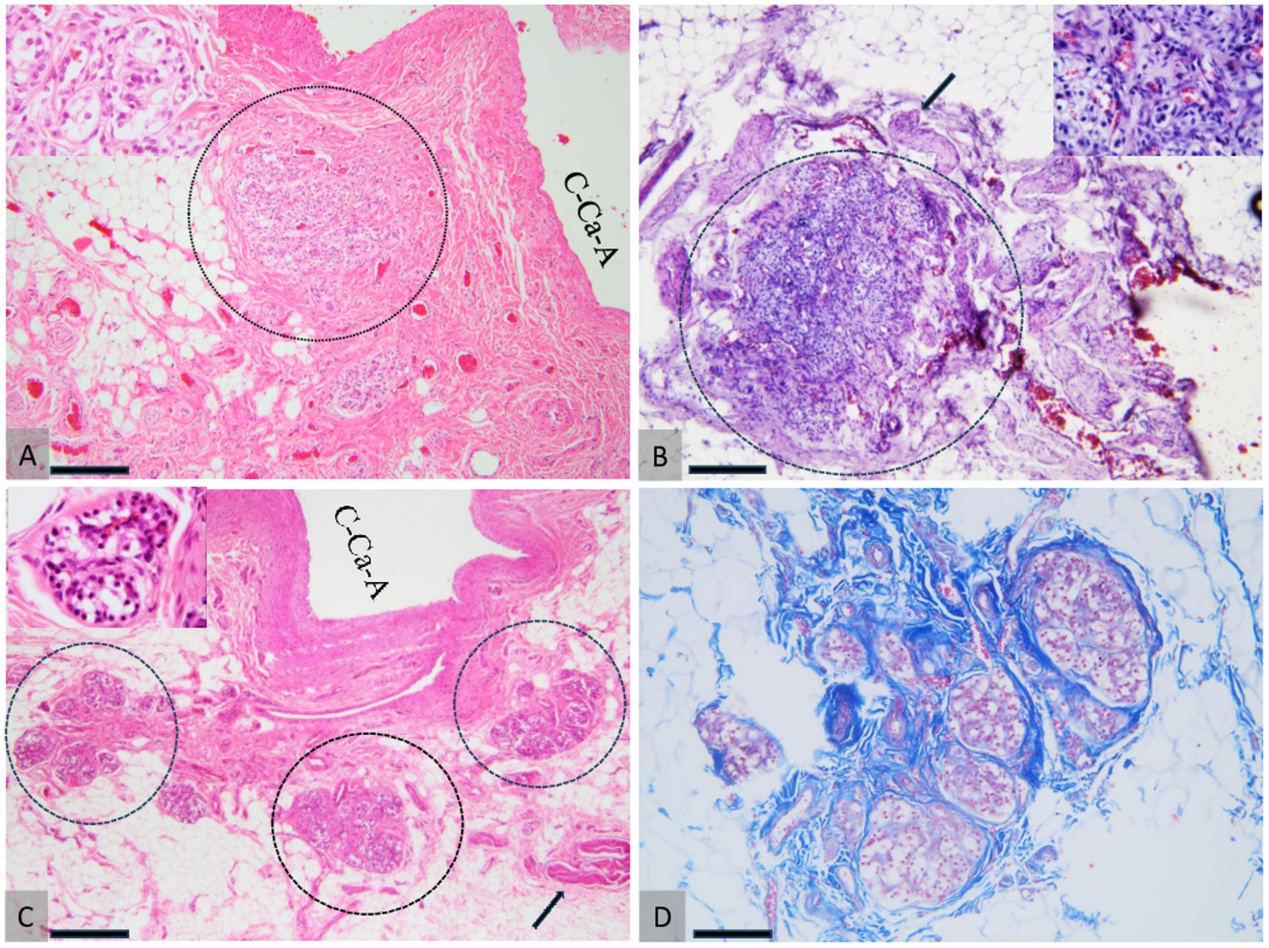
Comparative histology of the CB. Dog (A), cat (B), and ovine (C, D). (A) The CB in a dog, snowing tightly packed glomic clusters supported and demarcated by fibrous connective tissue, located near the wall of the common carotid artery (C-Ca-A). (B) CB in cat embedded in the adjacent adipose tissue, bordered by fibrous connective tissue. The arrow indicates the glossopharyngeal nerve, which innervates the CB. (C, D) CB in sheep consists of three-lobular structures (demarcated by the dotted circles in A) supported by high amounts of fibrous connective tissue (D) located in the proximity of the wall of the common carotid artery (C-Ca-A). The arrow indicates the glossopharyngeal nerve. H&E staining (A–C) and Masson’s trichrome (D). 20× scale bar = 200 μm (A–C) and 20× (D). 10× scale bar = 200 μm bar.

The position of CB is an excellent location in the carotid sinus. It presents an important function for cerebral blood flow and the compensatory mechanism that responds to fluctuations in arterial blood gas concentrations, particularly oxygen and carbon dioxide gas (22, 41). As tiny as this corpuscle may seem, it has one of the richest vascular networks in the body. It has been proven that if we compare the blood supply of the CB in the ratio with the human brain, which is (50 ml/min/100 g) (43, 44, 170), and tissues of the heart (80 ml/min/100 g) (45, 46), and other vital organs in comparison, we will notice that the blood supply of the CB is mainly enriched, for instance, in cats total blood flow, which is (1,410–2,000 ml/min/100 g) (46, 47), and in rabbits (700–1,200 ml/min/100 g) (48). It was reported by Clarke et al. (49) that in rats CB body and non-human primates, blood flow consists of 104 ml/min/100 g and (or 31 ml/min/100 g) correspondingly. The vascular irrigation was described clearly by Laure TD. and McDonald’s (4, 171). In accordance with their works, the blood flow of the CB consists of one or two glomic arteries, which are immediately divided into small arteries, capillaries, and venules. In the rat, blood irrigation takes root from a single artery called “CB-artery”—which takes root from the external carotid artery or occipital artery. The sanguine supply in dogs of CB derives from an occipital artery or the external carotid artery, sometimes from an ascending pharyngeal artery, it depends on the size of the animal and breed. In cats, the vascularization takes root from the occipital and pharyngeal arteries. In sheep and goats, irrigation is supplied by the occipital artery Then CB arteries and veins are divided into glomus venules and arterioles, creating a rich anastomoses network that pierces through the intercellular space (Table 3; Figures 1, 6) (50–52).

**Table 3:** Possible origins of CB blood supply in different species.

Species	Origin	Size	
*Homo sapiens*	Carotid bifurcation External carotid artery Internal carotid artery Ascending pharyngeal artery Vertebral artery Thyrocervical trunk	1.5–7 mm/12–18 mg	Heath et al. (40) Sarrat-Torres et al. (148) Ozay et al. (149) Muthoka et al. (150) Heath et al. (151) Nguyen et al. (152)
*Pan troglodytes*	Not known	0.8–1.3 mm/not known	Hansesn (153). Clarke et al. (49)
*Ovis aries*	Occipital artery	11 mm/10 mg	Sadik et al. (154) Najafi et al. (155)
Canislupus familiarris	Occipital artery, Ascending pharyngeal artery External carotid artery A muscle branch of the external carotid artery	1–3 mm/not known	Chungcharoen et al. (52)
*Felis catus*	Occipital ascending pharyngeal trunk Occipital artery Ascending pharyngeal artery External carotid artery	0.45–1.2 mm/2 mg	Chungcharoen et al. (52) Davis and Story (156) Ziemak et al. (157), Clarke et al. (49)
*Oryctolagus cuniculus*	External carotid artery Internal carotid artery Bifurcation	0.8–1.9 mm/not known	Chungcharoen et al. (52) Clarke and de Burgh Daly (24); Eken et al. (147)
*Cavia porcellus*	Not known	0.5 mm/0.8 mg	Clarke and de Burgh Daly (158); Docio et al. (159)
Ratus norvegicus	External carotid artery Occipital artery	0.4–0.8 mm/0.06 mg	McDonald and Larue (4) Habeck et al. (159) Unur and Aykan (160) Hess (161) Clarke and de Burgh Daly (158)
*Mus musculus*	Not known	0.4 mm/not known	Clarke and de Burgh Daly (158)

Adapted from Brognara et al. (147).

## 4 The pathology of the CB

Largely, the main pathology of the CB is represented by the oncological processes. There are isolated cases of non-neoplastic pathologies of the CB. Since the organ is very small, it can be involved in some adjacent pathological, inflammatory, hypertrophic, and septic processes.

Hypertrophy of CB was observed in humans with cystic fibrosis (a hereditary condition that impairs the lungs and digestive system by affecting cells responsible for creating mucus, sweat, and digestive fluids) and cyanotic congenital heart diseases. Changes in lobules, parenchyma, and stroma of CB have been observed. There was also an increase in the length and width of the lobules. However, the diameter of the cell cords and the size of the chief cells have not changed their normal volume (53–55).

The hypoplasia of CB was observed in a few postnatal rats, which were supposed to have induced hyperoxia. It was demonstrated that postnatal hyperoxia causes atrophy of the CB and CSN complex, resulting in a smaller number of chemoreceptor cells and fibers. CB volume decreased significantly in neonatal rats reared in 60% O_2_ (when the normal one is 21%) (56). This finding supports that postnatal exposure to hyperoxia causes significant and lasting atrophy of the carotid body (CB) and the carotid sinus nerve (CSN) complex in adult rats. This atrophy is evidenced by a marked reduction in CB size, a lower number of hypoxia-sensitive CSN fibers, and decreased dopamine (DA) synthesis and release in response to hypoxia. Despite these morphological changes, the survival of chemoreceptor cells and fibers in the hypoxic rats is sufficient to evoke normal increases in ventilatory frequency in response to hypoxia and hypercapnia if the hypoxic stimuli are intense enough. During chronic hypoxia, there is an increase in size and cell proliferation, which confirms the hypothesis of hyperplasia of CB in high altitudes (57, 58).

In rats, quantitative analysis revealed that the average volume of Type I cells increased significantly, from 320 μm^3^ in control rats to 1,120 μm^3^. In chronically hypoxic rats, hypoxia enhances the proliferation of Type I cells in the carotid body (CB). This proliferation leads to an increase in the size of the CB. It also influences chemoreceptor activity: Type I cells, which are chemoreceptors, respond to hypoxia by rapidly depolarizing their membranes, releasing neurotransmitters like ATP and acetylcholine. This triggers the activation of sensory neurons and initiates a reflex ventilatory response to improve oxygen supply. As a result, neurotrophic factors are observed to be expressed: hypoxia promotes the expression of tyrosine hydroxylase and synaptophysin in Type I cells, supporting their chemoreceptive functions (59, 60).

Wang ZY observed that hypoxia stimulates cellular growth while hyperoxia depresses it, yet both conditions depress CB chemoreceptor responses to acute hypoxia in neonatal animals. Differences in gene expression, particularly of neurotrophic factors and receptors, as well as the influence of reactive oxygen species on cellular function, likely play roles in these contrasting effects (60).

In some congenital pathology like coarctation of the aorta in humans (cardiac abnormality resulting in a birth defect in which a part of the aorta is narrower than usual) (61). In newborn children, an enlargement of CB was observed (hyperplasia of sustentacular cells) (62).

### 4.1 The involvement of the CB in neurogenic hypertension in humans and animals

The World Health Organization (WHO) states that hypertension is a significant health issue, contributing to an elevated likelihood of developing heart, brain, kidney, and other ailments. It represents a leading factor in premature mortality worldwide, affecting more than a billion people, approximately 1 in 4 men and 1 in 5 women of the population (63).

#### 4.1.1 Human’s neurogenic hypertension

In Carrey’s research, it was shown that 14% of patients with neurogenic hypertension are already resistant to modern treatment (64). In the last two decades, scientists have discussed different possibilities for finding antihypertensive solutions. One of the methods used to combat the symptoms of neurogenic hypertension (NH) was the unilateral ablation of CB. NH is often related to dysfunctions in neural control mechanisms that regulate blood pressure. The experiment was performed on rats with unilateral CB resection. As a result, a reduction of neurogenic hypertension was obtained, approximately by 55%, and mentions of the result for 6 weeks. Due to this experiment, the detailed and multifaceted understanding of the CB presents clinical and academic interests (65).

If we consider neurogenic hypertension from the perspective of the carotid body’s neuro-regenerative properties, future studies might explore the role of neurospheres, namely of self-renewing and multipotent stem cells within the carotid body (CB), particularly through the use of neurosphere assays under hypoxic conditions, as highlighted by Pardal et al. in their work “Glia-like Stem Cells Sustain Physiologic Neurogenesis in the Adult Mammalian Carotid Body 2007.”

Regarding tumorigenesis. The identification of carotid body stem cells suggests that disruptions in their homeostasis could potentially contribute to the development of chemodectomas, a type of tumor associated with the carotid body, particularly in individuals with chronic hypoxemia or high-altitude residents (66).

More research is needed to evaluate the long-term side effects of CB ablation and mortality in terms of time and parallel conditions. Following this idea, another important finding is a selective denervation of the CB. It was confirmed that this significantly improves autonomic, respiratory, and cardiac functions in a model of congestive heart failure (CHF). By reducing sympathetic neural activity, normalizing disordered breathing patterns, and lowering arrhythmia incidence, CB denervation addresses key pathological mechanisms in CHF. Targeting CB chemoreceptors through denervation could be a viable therapeutic strategy to enhance clinical outcomes in CHF patients. It was later demonstrated that the dysfunctions and overactivation of the autonomic system of CB induce sleep apnea. Due to this, the results obtained on the selective denervation show good results on CB, which improves respiratory, autonomic, and cardiac functions. These findings suggest that partial denervation can solve major problems in patients with sleep apnea, congestive heart failure, and arrhythmia (66, 67).

Certainly, future research must work on a molecular basis for reflexes’ sensitivity to the tonicity of afferent chemoreceptors. Lamptey, in a recent publication, described nanotherapeutics as a potential solution in the treatment of neurogenic hypertension on 22 January 2023. As far as it is known, not all therapeutics can cross this border, and this is where nanotherapeutics can come in (9, 67).

#### 4.1.2 Hypertension in dogs and cats

The systemic hypertension is a persistent increase in systemic blood pressure. It is important to note that, systemic arterial hypertension in dogs and cats is a secondary sign of the underlying disease, while in humans, in 90% of cases, the etiology of hypertension is unknown. Primary (idiopathic, essential) systemic hypertension is rare in animals and is a diagnosis of exclusion. The use of the term “idiopathic” also means that the cause of the disease may be a disease that is in the preclinical stage. In cases where the underlying disease is rare (e.g., adrenal tumors such as pheochromocytoma or adenoma), finding the cause of hypertension depends on the thoroughness of the diagnostic tests (68, 69).

Nowadays, there are no data that can confirm the presence of significant morphological changes in CB in clinical cases of hypertension in animals. However, CB has normal response and excitation of mediators in any hypertensive stages. The last data collected by the American College of Veterinary Internal Medicine (ACVIM) confirmed the extreme rarity of pheochromocytoma, which could be associated with hypertension. More common are chronic kidney disease, acute kidney disease, and hyperadrenocorticism (naturally occurring iatrogenically) (69, 70).

### 4.2 Neoplasia of CB and aortic body

According to the World Health Organization (WHO), paragangliomas are neuroendocrine tumors of extra-adrenal origin of the sympathetic and parasympathetic paraganglia. The term pheochromocytoma is reserved for tumors originating from chromaffin-producing cells, catecholamines, which correspond to the medulla of the adrenal gland (71, 72). Paragangliomas are a group of tumors that can develop in many regions of the body, such as the head area, neck, chest, and abdomen. Their origin is from the paraganglia, which in turn develop from primitive neural crest cells and is associated with nerve ganglia, structures belonging to the autonomic nervous system. From the point of view of cell morphology, both types (sympathetic and parasympathetic ganglia) are similar; both possess catecholamines but are quantitatively different. In humans, clinical studies have shown that the hypersecretion of catecholamines is only found in tumors originating from sympathetic paragangliomas (73).

#### 4.2.1 Incidence of the CB and aortic body tumors in domestic animals

The neoplasms of the carotid and aortic bodies are more frequent in animals, but the percentage is reversed for humans. These tumors are found especially in dogs and sporadically in cats and cattle. A high predisposition was observed in brachycephalic dog breeds, such as Boxers, Boston Terriers, Bullmastiffs, and Chow-Chows. Usually, these are animals older than 7–8 years, with a higher incidence in the female population. Approximately 65–70% of reported CB tumors still have AB tumors (74, 75). Aortic body tumors are the second most common cardiac tumor after hemangiosarcoma (76, 171) (Table 4).

**Table 4:** Reported case in domestic animal of aortic and CB tumor.

Animal	ABT	CBT	
Cat	Aortic body tumor Aortic body carcinoma	– –	(88, 99–101)
Mink	–	Carotid body tumor	(102)
Horse	Aortic body adenoma	–	(103)
Cow	Aortic body chemodectoma	– Carotid body tumor	(104, 105)

Tumors of the aortic bodies frequently appear in dogs, either as a single mass or as multiple nodules in the pericardial sac at the base of the heart. Sizes vary (0.5–12 cm), malignant paragangliomas being usually larger than benign paragangliomas. The incidence of these neoplasias is high in dogs over 6 years old but decreases with age of 15 years (77). Benign aortic body paragangliomas are either solitary or attached to the adventitia of the pulmonary artery and the ascending aorta or included in the adipose tissue between these two structures. They have a smooth surface, and on the section, the color is white, with the presence of red-brown areas. Big tumors have the potential to exert pressure on the atrial cavity or trachea, displaying multiple lobes and encircling the aorta at the heart’s base. Although the blood vessels are completely surrounded by neoplastic tissue, there are generally no signs of vascular constriction (78, 79).

**Figure 7:**
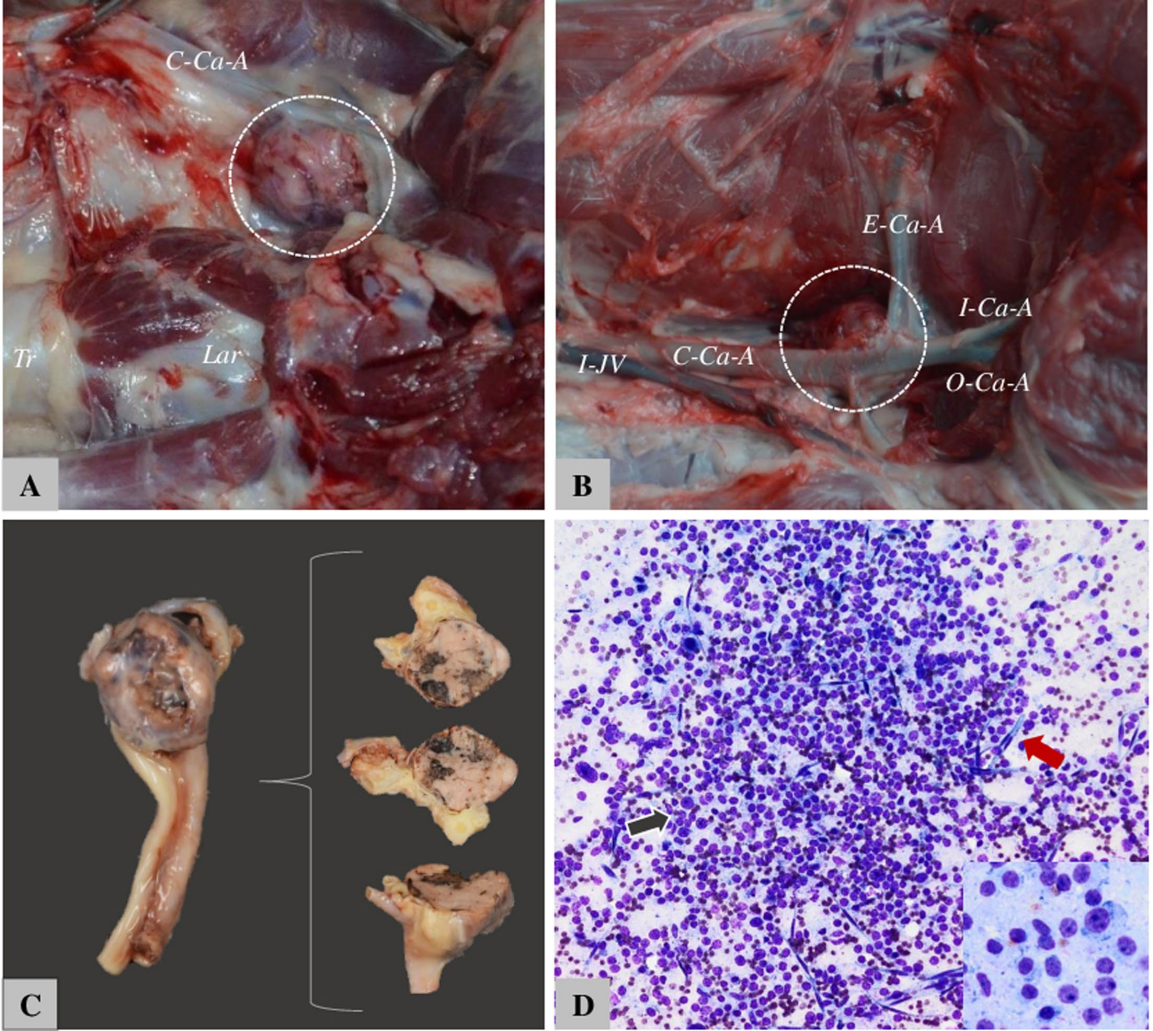
The appearance of CB tumors in dogs. (A) Boxer—12-year-old female, right CB. Tr. = Tracheia, Lar. = laringe, C-Ca-A. = common carotid artery. (B) Boxer—10-year-old male, C-Ca-A = Common carotid artery, I-Ca-A = internal carotid artery, O-Ca-A = occipital carotid artery, E-ca-A = external carotid artery. (C) Tumor situated on bifurcation of CA and sectioned for histology. (D) Fine-needle aspiration cytology from the carotid body tumor in dogs—nuclei are round to oval with anisocariosis, and variable degrees of pleomorphism.

CB tumors tend to be more malignant than aortic ones (Figure 7), Aortic body tumors in animals are not functional but cause identified conditions clinically due to compression of adjacent tissues, including cardiac decompensation as a result of the pressure exerted on the atrial cavity and the vena cava, both associated with tumors of large dimensions (75, 77). In addition, they might experience dyspnea, cough, vomiting, cyanosis, hydrothorax, hydropericardium, ascites, subcutaneous tissue edema (including the head, neck, and forelimbs), and passive liver congestion. The accumulation of serous or sero-sanguinolent fluid in the pericardial sac is the result of invasive tumor cells in the lymphatic vessels at the base of the heart or compression of the small vessels that irrigate the pericardial sac. The association in one patient of multiple neoplasia is common (up to 50% of dogs with tumors of the endocrine system), the most common tumor competitors being: testicular, ovarian, thyroid, parathyroid, adrenal, pituitary, and pancreatic (76, 77).

#### 4.2.2 Diverse pathologies of the carotid region: navigating through neoplastic and non-neoplastic conditions

**Figure 8:**
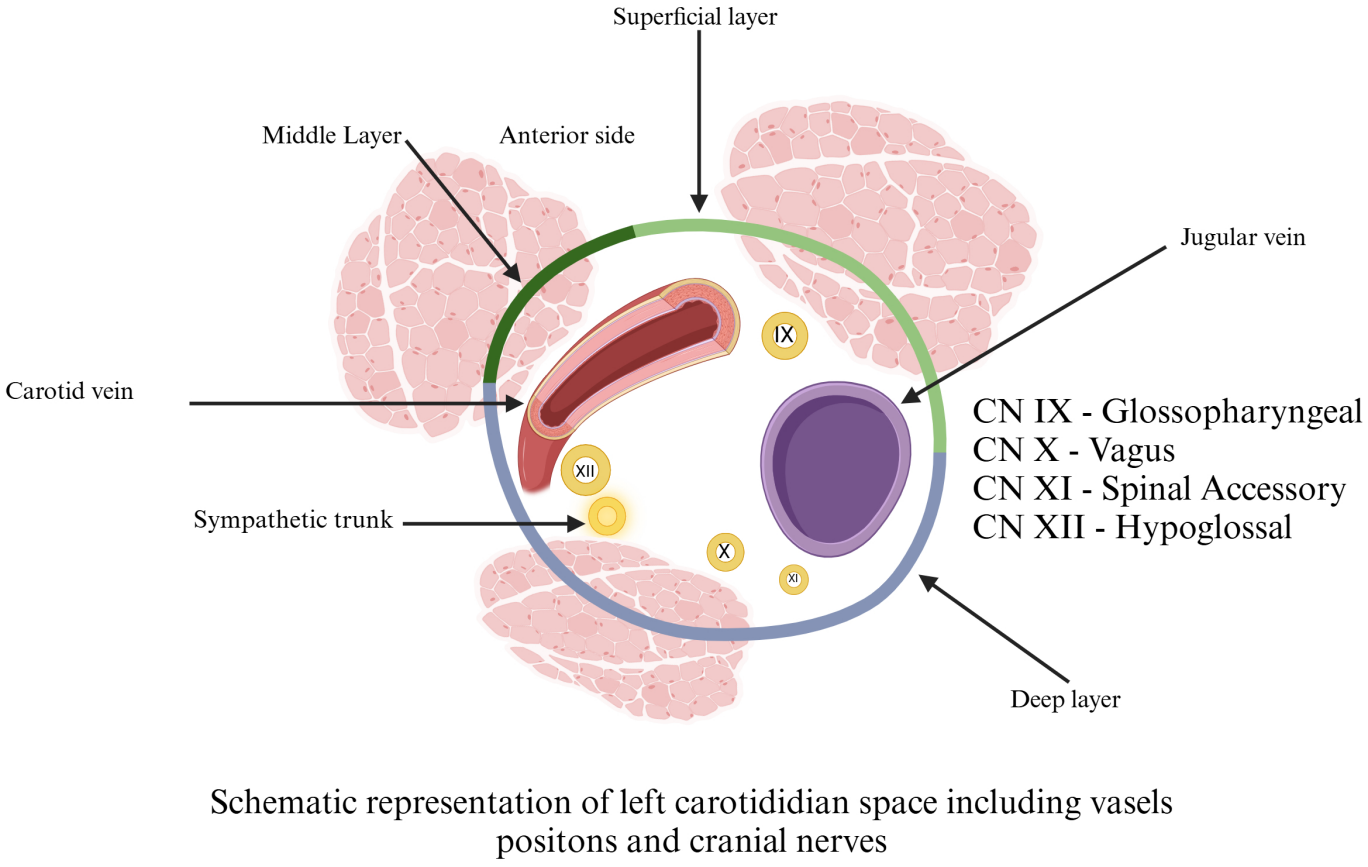
Schematic representation of left carotidian space, including vessel positions and cranial nerves. Cranial Nerve IX = glossopharyngeal. Cranial Nerve X = vagus. Cranial Nerve XI = spinal accessory. Cranial Nerve XII = hypoglossal. Adapted from Chengazi and Bhatt (68).

The pathology of the carotid region is extensive. In carotidian space (Figure 8), there are many macroscopic and microscopic changes in various organs of this region, including paragangliomas (such as CB tumors, glomus jugulare tumors, and glomus vagale tumors). Nerve sheath tumors (like neurofibroma and Schwannoma), and carotidian sheath meningioma. Non-neoplastic pathology of the carotid artery and internal jugular vein, carotid dissection and carotid pseudoaneurysm carotid thrombus, fibromuscular dysplasia, and jugular vein thrombus, pathology of the deep cervical lymph nodes chain, lipomas, and lymphomas (14, 27–29).

#### 4.2.3 Shamblin’s classification and potential adaptations: evaluating CB tumors

**Figure 9:**
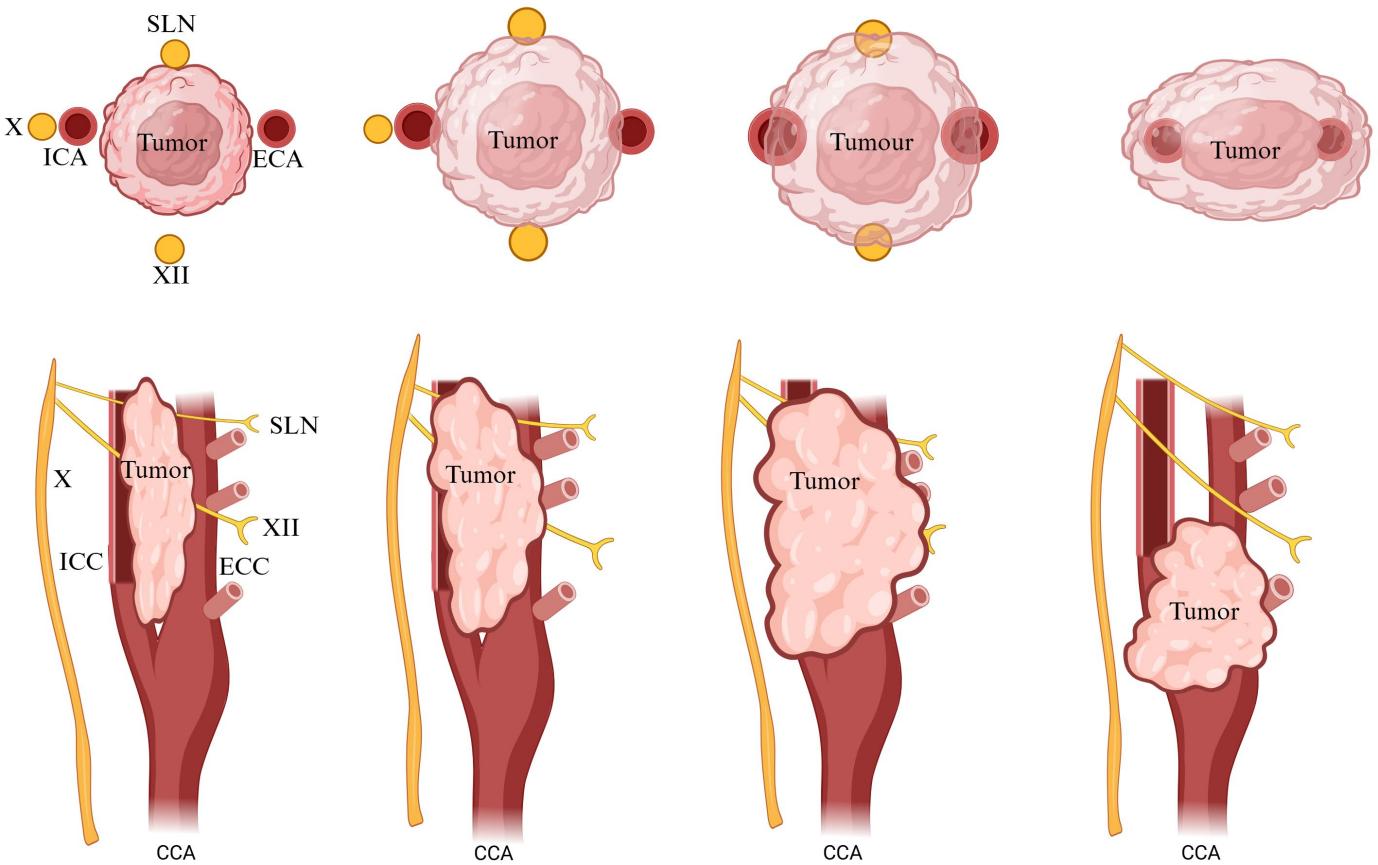
Classification of CB tumors. X, vagus, XII; hypoglossal nerve; ICA, internal carotid artery; ECA, external carotid artery; CCA, common carotid artery. Adapted and modified from Arya et al. (80).

Nowadays, Shamblin’s classification of CB tumors is used in human surgery. This is based on involvement and adherence to ICA and ECA. There is a schematic picture of CBTs into types I, II, and III. Type I tumors are small lesions that do not involve the carotid bifurcation. Type II tumors exhibit greater size and prominently spread apart the bifurcation, yet they do not fully surround the carotid arteries. In contrast, type III tumors are more extensive, enveloping the external or internal carotid arteries and often sticking to or integrating with the neighboring cranial nerves. However, later researchers proposed some modifications of Shambling’s to add one more type: type IIIb tumors that include tumors of any size that are intimately adherent to the carotid vessels. The oblique lines shown represent the X and XII nerves, which are related to the tumors (Figure 9) (80, 81).

### 4.3 Etiology of paragangliomas in animals and humans

In humans, paragangliomas are tumors originating from paraganglia cells, present in structures associated with the vegetative nervous system. As well as in humans, the origin and development of paragangliomas are similar. However, there is no solid genetic confirmation of like genetic mutation in humans. It is assumed that the increased incidence of neoplasia of these chemoreceptors in brachycephalic breeds is associated with chronic hypoxia. In a recent study, it was once reported that a somatic mutation in the SDHD gene, namely a c.365A > G (p.Lys122Arg) substitution located in exon 4, could be associated with dog paraganglioma (82). But this unique case necessitates more confirmation. Although in humans, genetic studies have shown that 87.1% of cases of paragangliomas are associated with mutations in the SDHD gene (83–85).

#### 4.3.1 The genetical approach of CB tumors in humans and dogs

##### 4.3.1.1 SDH mutations in humans

In humans, genetic studies have confirmed the involvement of mutations in the occurrence of this tumor, with 87.1% of benign tumors produced by germline mutations of the gene encoding the succinate enzyme dehydrogenase subunit D (SDHD) (83). The genes that have previously been associated with paraganglioma in humans are NF1 (causing neurofibromatosis type 1), RET (endocrine neoplasms multiple types 2a and 2b) (73), VHL (von Hippel–Lindau syndrome), SDHB, SDHC, and SDHAF2 neurofibromatosis type 1 appears because of a mutation in the NF1 gene located on chromosome 17q11. “Encodes the protein neurofibromin1, with a function in cell signaling, cell growth, differentiation, and survival” (85, 86). Paragangliomas associated with neurofibromatosis are very rare. In a study that included 809 paragangliomas in the area of the head and neck, no mutation in the NF1 gene was identified (87).

Mutations in the RET gene (proto-oncogene) located on chromosome 10q11 cause type 2 multiple endocrine neoplasia (MEN) syndromes. The RET gene encodes a receptor for tyrosine kinase and functions as a receptor for extracellular signaling molecules for glial cells. In MEN syndrome type 2b, the incidence of occurrence of head and neck paragangliomas is 0.1% (88), and for other extra-adrenal paragangliomas, the incidence is 5% (89, 90).

Von Hippel–Lindau is a hereditary neoplastic syndrome caused by mutations in the VHL gene located in chromosome 3q25–26. The VHL gene encodes a subunit of the key enzyme complex responsible for the degradation of hypoxia-inducible factor (HIF), subunits 1α, 2α, and 3α. The syndrome is autosomal dominant [143]. Paragangliomas of the head and of the neck are found in <1% of cases with von Hippel–Lindau syndrome (89–91).

The enzyme succinate dehydrogenase subunit D was the first protein identified, with a role intermediate in metabolism, and also the first mitochondrial enzyme to be linked directly to tumorigenesis (92). This enzyme is encoded by the SDHD gene, located in chromosome 11q23. Patients in whom mutations in this gene have been identified, from the point of clinically, are associated with the appearance of benign head and neck paragangliomas, metastases being very rare. In addition, in these patients, there is a fairly high risk of developing multiple paragangliomas and pheochromocytoma (93).

The SDHC gene located on chromosome 1q23, which encodes the C subunit of the succinate enzyme dehydrogenase, was identified as a tumor suppressor gene. Mutations in this gene are associated with the appearance of benign paragangliomas in the head and neck (94).

In 2001, the SDHB gene encoding the iron–sulfur catalytic subunit of succinate dehydrogenase, located on chromosome 1q35–36.1, was also associated with paraganglioma. It acts as a tumor suppressor gene and has been shown to be the dominant cause of human paraganglioma in many parts of the world (95).

##### 4.3.1.2 SDHD mutation in dogs

One of the theories of the etiology of paragangliomas suggests that chronic hypoxia causes cell hyperplasia (96), which results in the transformation of neoplasticity of chemoreceptor cells (97). The risk of tumor formation is 3.8 times higher for spayed females than for non-spayed ones, but not for males, there is a significant difference between neutered and uncastrated males (98).

In bulldog breeds there is a high risk for aortic tumors, a fact supported by a retrospective study of individuals belonging to these races. Genetic predisposition, aggravated by stimulation of high pCO_2_ and low O_2_, results in high tumor frequency. Dogs with aortic body tumors show signs of respiratory acidosis with low O_2_ and elevated CO_2_ with normal pH. A large aortic tumor can cause acidosis, but a study in which dogs with very small tumors were also monitored showed respiratory acidosis. The theory does not include all brachycephalic breeds; for example, the Pug or Pekingese does not have a higher incidence than other non-brachycephalic breeds (68–97, 99–110).

In a recent study, starting from human research on the genetic etiology of the occurrence of paraganglioma and its hereditary character, the possible occurrence of some mutations in the SDHD and SDHB gene exons in two Boston Terrier and Golden Retriever dogs with carotid paraganglioma was investigated. The results consisted in the identification of two somatic mutations in the Boston Terrier in the SDHD gene: a silent type mutation in exon 3 (c.291G > A (p.Ala97Ala)) and a type missense in exon 4 (c.365A > G (p.Lys122Arg)). In exon 2 of the SDHD gene in the Golden Retriever, a silent type mutation (c.156A > T (p.Ser52Ser)) was identified. It was not identified SDHB gene mutation (82). The theory regarding the link between gene mutations that encode succinate dehydrogenase subunits and the development of neoplasia requires stabilization of hypoxia-inducible factor (HIF), which is normally induced by hypoxia. Impoundment of excess succinate due to mutations stabilizes the HIF 1α subunit even at a normal level of blood oxygen (pseudohypoxia) by inhibiting prolyl hydroxylase, an enzyme whose role is to reduce the hypoxia factor. The stabilization of HIF 1α results in the transcription of the genes that encode its endothelial growth factors and other genes possibly involved in the development of neoplasia. Another proposed mechanism is the inhibition of apoptosis and the generation of reactive oxygen species (111).

#### 4.3.2 Diagnostics of CB tumors in animals

##### 4.3.2.1 Clinical appearance

Clinically, the diagnosis of aortic body tumors and cardiac tumors, in general, is a complex task due to their rarity. Aortic body tumors, particularly the carotid body tumors, are infrequent and more often malignant. However, recent findings have presented an atypical case of recurrent carotid body carcinoma in a young adult Chihuahua, highlighting the unique and complex nature of these diagnoses (54, 112).

Small Carotid Body Tumors (CBTs) 1–2 mm at routine control are often not detected due to their subtle nature and lack of significant symptoms. Large CBTs greater than 7 mm, can be observed through palpation during a physical examination. Aortic body tumors are usually detected during routine echocardiography. The clinical signs are non-specific and indicate only a cardiac condition. In the case of CB, the most common clinical signs are muscle atrophy of the head, neck pain, dysphagia, cough, and anorexia (55, 113–116). Tachycardia, tachypnea, hypotension, increased capillary refill time, ascites, cyanosis, and lethargy could also be observed. CBTs represented a palpable mass of variable size ovoid spherical at the level area of the carotid bifurcation. The mass is typically mobile laterally but not vertically due to its attachment to the carotid artery. As the tumor enlarges, it can compress nearby cranial nerves, leading to various neurological deficits. Respiratory symptoms are also common, particularly apnea symptoms. Many CBTs are malignant, unlike aortic body tumors, and they occasionally spread to regional lymph nodes, lungs, liver, pancreas, bones, and kidneys (55, 115–117).

The neural symptoms are very rare. However, they can be observed by irritation or loss of nerve function. These symptoms depend on the tumor’s location related to the nerve trunk. If the tumor is located in the center of the nerve, then the compression of the fibers is more pronounced, and the neurological manifestations are more observed; at the marginal location of the tumor, compression of the nerve is less significant and, accordingly, poorer neurological symptoms (117).

Advancements in imaging techniques, such as computed tomography (CT) and magnetic resonance imaging (MRI), have significantly improved the diagnosis and management of CBTs. These imaging modalities provide detailed information about the tumor’s size, location, and relationship to surrounding structures, aiding in surgical planning. Angiography can be useful in determining the vascular supply of the tumor and planning surgical resection, as well as identifying multiple paragangliomas in patients with familial syndromes (55, 116, 117).

Recently, a paraneoplastic syndrome of Paraneoplastic Limbic Encephalitis associated with a unilateral CB paraganglioma was documented in a human patient (117). Paraneoplastic syndromes associated with CBTs were not documented in veterinary medicine.

##### 4.3.2.2 Histopathological and immunohistological structure of paragangliomas

Despite the already existing methods of visual diagnostics, histological analysis remains the gold standard. The histopathological and immunohistochemistry (IHC) diagnosis is always based on the samples obtained from the follow-up biopsy or necropsy examination. IHC provides high specificity and sensitivity in detecting specific proteins within cells and tissues.

Paragangliomas, as we indicated above, are tumors with neuroendocrine origin. Accordingly, molecules such as tyrosine hydroxylase and GFAP (glial fibrillary acidic protein) mentioned below in paragraph I.2. can be used in immunohistochemistry. However, TH has several limitations since not all paragangliomas are positive for this marker (118, 119). It still provides valuable information about the biosynthetic capabilities of the tumor cells and their differentiation status in dopamine-screening tumors. However, Cg-A staining and neurospecific enolase could offer supplementary diagnostic value (120, 121).

In the veterinary literature, chromogranin A, which is contained within secretory granules, offers a more straightforward and consistent staining pattern that aids in diagnosing neuroendocrine tumors. Whereas (TH) can be used to differentiate them from thyroid follicular and C-cell tumors (118, 120–122).

Consequently, chromogranin A, TH, and neuron-specific enolase are employed in the immunohistochemical evaluation of paragangliomas because they offer reliable, specific markers of neuroendocrine differentiation and origin. Regarding S-100, protein is a reliable marker for identifying sustentacular cells. These cells provide structural support to chief cells and play a paracrine role. Protein S-100 can identify the cells that influence the tumor microenvironment and play a significant role in the tumor’s structural and functional dynamics (123, 124).

Accordingly, identifying these cells is crucial for predicting tumor behavior. Using a single marker for immunohistochemistry (IHC) is insufficient for accurately predicting the clinical behavior of paragangliomas due to their cytologic heterogeneity. Paragangliomas consist of two distinct cell types: chief cells (type I) and sustentacular cells (type II), each with unique markers (118, 122, 123).

**Figure 10:**
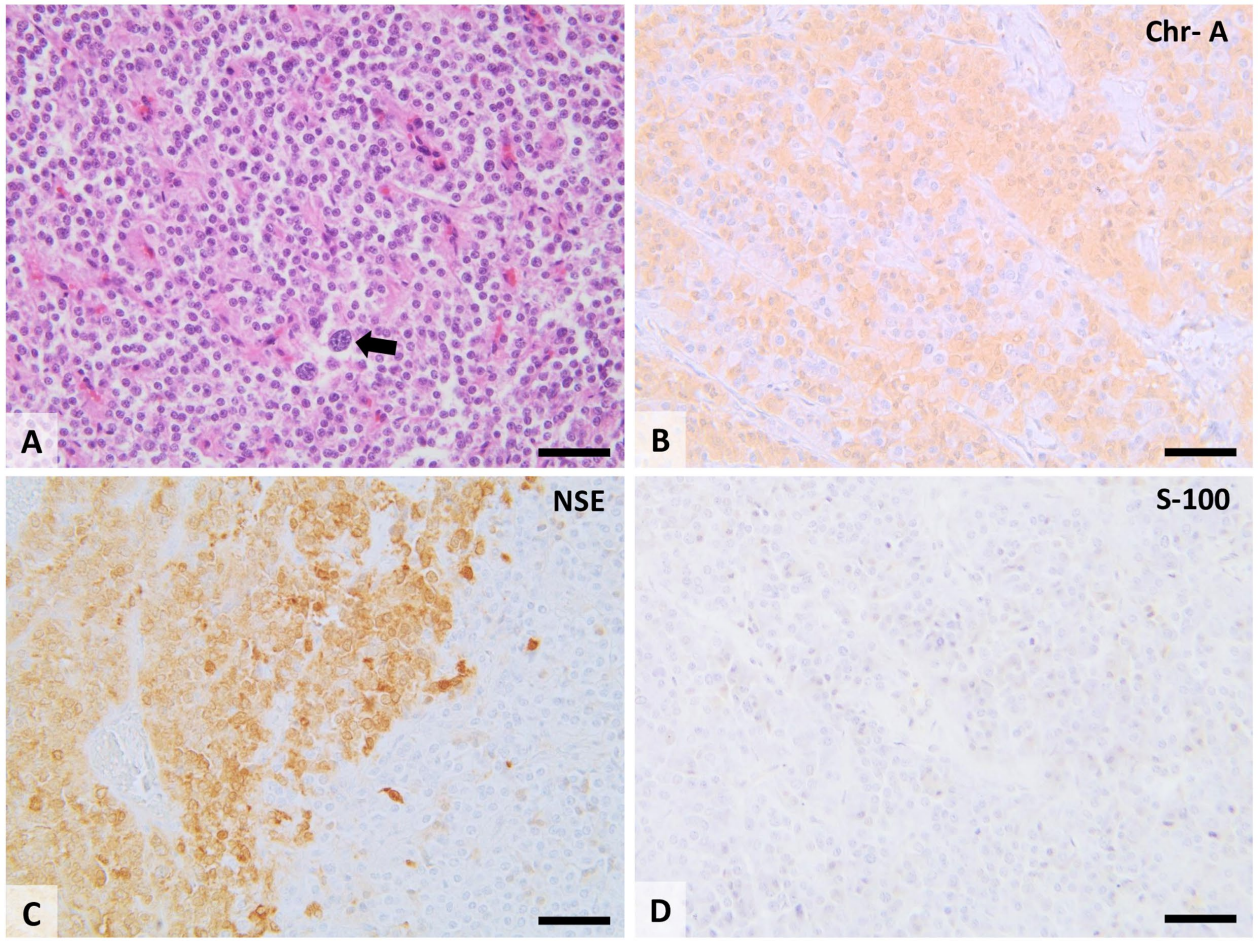
Histopathological features of the CB chemodectoma. Tumors consist of tightly packed clusters and lobes of round to polygonal cells with moderate cell polymorphism, separated by a small amount of fibrovascular stroma. Some tumor cells have karyomegaly and cytomegaly (A; arrow). Tumoral cells show extensive cytoplasmic immunolabeling for chromogranin A (B; Chr-A), and neuron-specific enolase (C; NSE) and show no immunolabeling for protein S-100 (D). 20× scale bar = 200 μm.

Histologically, paragangliomas are presented as a dense population of medium-sized, round cells, or polygonal, surrounded by a thick fibrous capsule. Tumoral cells form compact, spheroid nests of variable sizes (“Zellballen” arrangement), surrounded by fine, usually well-vascularized, fibrous tissue. The malignant paragangliomas present the following characteristics: pleomorphism, anisocytosis, anisokariosis, even megalocytosis and megalocariosis; a large number of cells can be observed in mitosis and cell invasion in the blood vessels and capillaries (Figure 10).

The cytoplasm of tumor cells varies from the point of view of tinctoriality according to the number of secretory organelles and stored granules. The intracytoplasmic presence of hormone secretory granules can be observed under the optical microscope only using Grimelius silver-based stains or Churukian-Schenk staining (118, 122). Ultrastructurally, the mitochondria within the paraganglioma cells can be observed arranged parallelly to the rough endoplasmic reticulum. In addition, the Golgi apparatus, with prosecretory granules, is highlighted in the cytoplasm. Paragangliomas were originally classified based on their affinity for staining by chrome salts (chromaffin reaction). This reaction suggests a sympathetic origin or production of catecholamines. Tumors that have their origin in the parasympathetic nervous system are referred to as non-chromaffin paragangliomas or chemodectomas. The chromaffin reaction does not allow a clear separation of these tumors because to some extent all paragangliomas produce catecholamines. Especially in the case of weakly malignant neoplasms, differentiated tumors can acquire new secretory capacities (98, 116).

## 5 Discussion

A major breakthrough in science is expected soon because, over the past few decades, quite a few tools for scientific researchers have been accumulated. Such tools as nanotherapy and artificial intelligence. The transition of modern scientists to the use of artificial intelligence helps in the synthesis of information. It is certainly becoming easier. The integration of AI in veterinary research, exemplified by tools like ChatGPT, can significantly enhance the comparative study in anatomy, physiology, pharmacology, etc. By automating data analysis and facilitating the comparison of anatomical and physiological characteristics, AI can help researchers identify patterns and variations that are critical for understanding the functional nuances. Artificial intelligence is a further step forward. It happens that the evolution of clinical decision-making and diagnostics in oncology saves crucial time for patience. However, all these instruments raise important questions about data and privacy, particularly when dealing with genetic information. Additionally, the potential for AI bias—stemming from the data on which these tools are trained—must be carefully managed to ensure that diagnostic and treatment recommendations are accurate and equitable. Finally, the integration of AI into clinical decision-making should complement, rather than replace, the expertise of veterinary professionals, ensuring that the human–animal bond and the nuanced understanding of individual cases remain central to veterinary care.

It’s important to acknowledge the limitations of our study. In any literature review, there are always gaps that cannot be filled by navigating online libraries and platforms used by PubMed, Web of Science, Springer Link, Google Scholar, and others. Many records are not written in English, and some of them are still being discovered by historians and archeologists despite not being digitized. This is crucial for understanding the logic of discoveries and their sequence, as in the case of De Castro, who made a huge number of discoveries in his area and dedicated practically all his life to research of CB, but the Nobel Prize was won by the person who collaborated with him just twice. Although nowadays it is easier to navigate scientific libraries and platforms, it will always be hard to estimate the probability of errors and bias in used and cited literature (125–128).

## 6 Conclusion

In conclusion, our comparative exploration of the CB in domestic animals has provided valuable insights into its anatomical, physiological, and pathological aspects. This review has examined the histology of normal and pathological CB and shared characteristics and unique distinctions across different species. A complete understanding of the carotid body’s function, innervation chain, and redirection of impulses provides an opportunity to deal with neurogenic hypertension and sleep apnea, the causes of which, as we know, are not fully established. The elucidation of chemoreception and the genetic core basis of CBTs provides valuable insights into the fundamental mechanisms of the hypoxic response and tumorigenesis. The interplay between CB function and systemic diseases like heart failure and neurogenic hypertension opens new therapeutic possibilities, highlighting the significance of continued research in this domain. Future studies should focus on further characterizing the molecular pathways involved in CB-mediated responses, exploring targeted interventions, and integrating new therapeutics to improve clinical outcomes in related diseases.

While neuroendocrine cells may appear to operate through a seemingly straightforward system, our analysis reveals variations in blood supply and predispositions to neoplasia across species. Notably, the pathology of CB predominantly involves neoplasias, which are most commonly observed in dogs and humans and less frequently in other animals. This suggests a tumorigenesis hypothesis that may be associated with either genetic susceptibility or factor HIF.

At the current moment, we do not have enough comprehensive and definitive evidence to confirm the uniformity or consistency in genetic traits across the studied subjects or populations. This lack of conclusive data means that there may still be significant genetic variations or nuances yet to be discovered or understood in the context of our research.

Up to the present time, researchers and veterinarians have not identified any hereditary conditions confirmed genetically in dogs or cats that closely resemble the multiple endocrine neoplasia (MEN) syndromes observed in humans. The small evidence and relevant number of findings in dogs and cats suggest that their genetic predisposition or disease mechanisms might differ from those in humans. To comprehensively discern the parallels between the MEN syndrome-related involvement of chemoreceptors such as AB and CB observed in both animals and humans, it remains imperative to accumulate and analyze more data to guide future scientific investigations.
